# Are Sex Offending Allegations Viewed Differently? Exploring the Effect of Offense Type and Conviction Status on Criminal Stigmatization

**DOI:** 10.1177/10790632231154168

**Published:** 2023-01-30

**Authors:** Craig A. Harper, Philip N. S. Rumney, Deborah A. Sackey

**Affiliations:** 1 NTU Psychology, 6122 Nottingham Trent University, Nottingham, UK; 2 34632 Leicester De Montfort Law School, De Montfort University, Leicester, UK

**Keywords:** sexual offending, crime accusations, stigmatization, public perceptions, criminal justice policy

## Abstract

Attitudes towards individuals with sexual convictions play a major role in the formation of legislative action, including sentencing policies and registration and notification procedures. However, there is little research about stigmatization directed at those who are accused of such offenses prior to conviction. In this work we explored this gap by comparing stigmatization (e.g., a desire for social distance, and negative personality attributions) towards people accused of a range of crimes (sexual, violent, and acquisitive), and whether this was further impacted by whether or not allegations led to a conviction. We recruited 403 community-based participants for a between-subjects experimental survey. We found support for the conclusion that people accused of and convicted for sexual offenses are more heavily stigmatized than allegations related to other crime types, and especially so when allegations involved child victims. Stigmatization took the form of greater levels of support for police-initiated notifications about allegations before conviction, increased desires for social distance, and attributions of negative personality traits. We discuss the theoretical and applied implications of these findings in relation to stigma research and issues related to anonymity for those accused of sexual offenses.

## Introduction

Attitudes towards individuals with sexual convictions have been widely studied and play a major role in the formation of legislative action, the design of treatment, and the social dynamics related to rehabilitation and community re-entry (Göbbels et al., 2012; Harper et al., 2017; Lowe et al., 2019). However, there is a lack of data that directly compares attitudes towards individuals with sexual and non-sexual convictions. This means that we are unsure as to whether there are specific issues (e.g., the nature of the harm caused by sexual crime, or its typically gendered nature) that drive social views about sexual (vs. other) crimes. At the same time, there are ongoing legal discussions about the ethics and practical utility of disclosing suspect identities prior to trial due to the high levels of stigma that accompany allegations of sexual offending (Cubellis et al., 2018). In this paper, we start to address some of these issues by exploring the stigmatization of those accused of sexual offenses, exploring public support for community notification of allegations of criminality by the police to local communities (e.g., to the media, employers, families), and understanding whether known stigmatization levels are contingent on the outcome of a trial. We also explore whether stigmatization is ameliorated by a public acquittal (via a not guilty trial verdict) of the allegation.

### Understanding Attitudes About Sexual Crime

An attitude is defined as a “psychological tendency that is expressed by evaluating a particular entity with some degree of favor or unfavor” (Eagly & Chaiken, 1993, p. 1). According to Breckler’s (1984) conceptualization, attitudes are comprised of three distinct yet inter-related components, known as the ‘ABC’ model:

1. Affect; that is, the intuitive or visceral emotional response to an attitude object;
2. Behavior; that is, the actions and behavioral intentions towards an attitude object. This can include both interpersonal actions and policy preferences;
3. Cognition; that is, the beliefs and stereotypes held about an attitude object, or the attributions made about it.

Among forensic psychological research, attitudes towards specific offending populations have been explored. None have been studied as much as individuals with sexual convictions, with most research finding negative social views at all three levels of Breckler’s (1984) conceptualization. At the affective level, people tend to associate the ‘sex offender’ label with negative emotions on an automatic or heuristic basis (Harper et al., 2017; Harris & Socia, 2016; Malinen et al., 2014; Viki et al., 2012). One hypothesis is that such automatic responses stem from associations (or attributions) made about this population due to indirect contact via sensationalist and emotive media reports (Greer, 2012; Harper and Hogue, 2015b, 2017; King & Roberts, 2017; Malinen et al., 2014). Such beliefs associated with skewed media reporting include believing individuals with sexual convictions are susceptible to high rates of re-offending, represent a homogenous population with a discrete number of core characteristics, and predominantly attack children or strangers (Galeste et al., 2012; Harper & Bartels, 2018; Harris et al., 2009). This not only contradicts officially recorded and empirically supported re-offending rates (Hanson et al., 2018; Sample & Bray, 2003, 2006; Tewkesbury et al., 2012), but these beliefs elevate fears related to sexual victimization and exacerbate a self-perpetuating cycle of negative public attitudes and emotive media reporting (Greer, 2012; King & Roberts, 2017).

A combination of these affective and cognitive processes leads to behavioral effects that contribute to social isolation among individuals with sexual convictions, as well as punitive legislative action. One such action is public notification of former offense status, and the use of ‘sex offender’ registries. Though there are possible theoretical benefits for registration (e.g., the ability for law enforcement to locate known individuals with offense histories upon their release from prison), there are worries about its efficacy and execution (for a review, see Levenson, 2016). Specifically, registration treats individuals who have sexually offended as a homogenous group despite intra-group variation (Ricciardelli & Moir, 2013; Sample & Bray, 2006; Vásquez et al., 2008; Zgoba & Bachar, 2009). Such measures posit sexual offenders are dangerous individuals who require prolonged periods of monitoring which works to further confirm the notion of susceptibility to re-offending (Kernsmith et al., 2009; Lowe et al., 2019; Willis, 2018). It is this notion of non-transformability that has also been linked to the generation of social hostility towards individuals convicted of sexual crimes (Pickett et al., 2013; Sample & Bray, 2006; Willis, 2018), with registration being seen by many members of the public as a functional form of social control (Dijker & Koomen, 2007; Levenson et al., 2007; Tewksbury, 2012). Registration procedures that lead to public disclosure of an offender’s history (such as those related to sexual offending in the US, and the Child Sex Offender Disclosure Scheme in England and Wales) can have substantial effects on the lives of individuals who are subject to them. These include difficulties in relation to gaining employment, and finding secure housing, as well as forming and maintaining close personal and intimate relationships (Evans & Porter, 2015; Levenson & Cotter, 2005; Socia, 2011; Zgoba et al., 2009). For example, Clark (2007) reported data on how landlords are less likely to rent their property to somebody with a history of sexual convictions (see also Evans & Porter, 2015). When accompanied by residency restrictions that are typically embedded in registration and license conditions (Levenson & Cotter, 2005; Zgoba et al., 2009), these difficulties can prevent individuals living near to family members or other sources of social support. When exploring the desistance literature (i.e., work looking at how and why people move away from patterns of criminal behavior), having access to social support and opportunities for housing, work, and relationships are all theoretically associated with lower odds of re-offending (Harper et al., 2017; Willis et al., 2010, 2013b). Thus, influencing public attitudes in a positive direction could be important to the broader social aim of reducing reoffending and preventing further victims of sexual harm (for a review and theoretical framework of desistance from sexual offending, see Göbbels et al., 2012).

Stigma can be considered a form of negative attitude, and is typically described in terms that relate to cognitive (i.e, attributions being made about a person as a result of one aspect of their identity) and behavioral (i.e., actions directed towards a person as a result of one aspect of their identity) domains. For instance, Goffman’s (1963) classical conceptualization of stigma frames this as being related to “an attribute that is deeply discrediting” (p. 3), inferring that it relates to the kinds of attributions that are made about a person’s moral character. Relatedly, Corrigan et al. (2008) cite stigma as being comprised of stereotypes, prejudice (both cognitive processes), and discrimination (a behavioral manifestation of negative views). The levels of stigma directed towards those with criminal convictions is generally high, but is intensified when the nature of an individual’s criminal history is sexual (Ricciardelli & Moir, 2013). It is also perceived by those with sexual conviction that the stigma attached to their offending is harder to overcome in the long-term than the stigma surrounding those with other offense types (Mann et al., 2021). Ricciardelli and Moir (2013) attributed this distinction to people perceiving those with sexual convictions as being more socially deviant than other groups, having violated sacred values by preying on the innocent and weak. Although this may be true in some cases, this stereotypical view of sexual offending is not true of all sex crimes. In a theoretical piece about this notion, Harper and Harris (2017) suggested that intuitive moral foundations related to the protection of innocent or virtuous groups may play a role in shaping social responses to sexual crimes. Similarly, Harris and Socia (2016) suggested that the ‘sex offender’ label is itself associated with various connotations that are activated heuristically (that is, automatically; see also Harper & Bartels, 2018; Harper et al., 2017; King & Roberts, 2017). Given the emotional coverage of sexual crime within the news media (Harper & Hogue, 2015a, 2017), it is likely that such heuristic processes are not involved to such a degree when people are making decisions about those involved in non-sexual offenses.

In one study, more negative attitudes (operationalized as support for more punitive punishments and less belief in rehabilitation) were reported in a sample of students in response to individuals with sexual convictions, as compared to the views expressed about those with non-sexual convictions (Rogers & Ferguson, 2011). However, no research appears to have been conducted that directly compares attitudes towards individuals with sexual and non-sexual convictions using general public or community samples. That said, similar trends have been found within prison settings, where those who are incarcerated after committing sexual offenses face high levels of stigma, even from those with other serious (though non-sexual) offenses on their records (Mann, 2016; Levins & Crewe, 2015; Ricciardelli & Moir, 2013; Tewksbury, 2012).

### Is Stigma Limited to Those Convicted?

Although most of the research published into attitudes towards sexual crime has focused on views about those with convictions, there is some evidence to suggest that the stigmatization of sexual offending is so pervasive that it extends to those who are merely suspected of committing offenses (Cubellis et al., 2018). In factorial analyses looking at support for various policies, those with sexual convictions have been more stigmatized than those receiving government welfare, the working poor, and homeless groups, as well as when compared to people with non-violent drug offense histories. For example, Dum et al. (2017) reported how people were less likely to support housing-related policies for people with convictions for sexual offenses (comparative to all the above groups), even when such a policy came at no cost to themselves. Similar data comparing support for residence restrictions for those with sexual convictions and drink driving offenses were reported by Levenson et al. (2012). However, these kinds of analyses do not directly compare the effects of being *accused of* a sexual crime (without being convicted) to being *convicted of* a sexual crime in relation to the experience of social stigma. As such, discussions around stigma and criminality raise important questions of public policy.

Currently, in England and Wales police forces are discouraged from disclosing the identity of criminal suspects prior to charge unless there is good reason, such as the prevention of crime (College of Policing, 2021). Further, identifying those who are the subject of criminal investigation may, in some limited circumstances, breach a legal right to privacy (Richard, 2018), although generally “privacy rights … are … not accorded greater weight than freedom of expression, when open justice and media freedom come into play” (McGlynn, 2011, p. 214). Guidance has been issued by the College of Policing to police forces which states: ‘[s]uspects should not be identified to the media (by disclosing names or other identifying information) prior to the point of charge except where justified by clear circumstances e.g. a threat to life, the prevention or detection of crime or a matter of public interest and confidence’ (College of Policing, 2021, para. 3.2). This is reflected in current police practice and it is rare for suspects to be named. In terms of those convicted of offences, the police in England and Wales have limited common law powers (R v Chief Constable of North Wales Police, 1998) to disclose the criminal history of offenders. The police also possess a statutory power under s.140 of the Criminal Justice and Immigration Act 2008 (amending earlier legislation), that enables a force area to disclose the criminal history of child sex offenders to the public. There is also a power to disclose non-criminal behavior where an individual poses a risk, and there is a pressing need to disclose to the public (e.g., an employer) in order to prevent harm (R (on the Application of A), 2010).

In England and Wales, there has been debate over whether those accused of rape or other sexual offenses should have their identity legally protected until charge or conviction (Rumney & Fenton, 2013). One of the arguments made in support of this view relates to the stigma and reputational damage that is said to be caused by sexual offence allegations, particularly where they attract media attention (Henriques, 2016; Leveson, 2012). In 2003, the UK Parliament’s Home Affairs Committee recommended anonymity for sexual offense suspects up to the point of charge. The committee argued that the stigma suffered by those accused of sexual offenses:

fall ‘within an entirely different order’ to most other crimes. In our view, the stigma that attaches to sexual offences - particularly those involving children - is enormous and the accusation alone can be devastating. If the accused is never charged, there is no possibility of the individual being publicly vindicated by an acquittal (Home Affairs Committee, 2003, para 76)

This recommendation, however, was not implemented by Parliament. This decision is likely related, in part, to the potential benefits of being able to name suspects on sexual offense cases. Such benefits are said to include providing victims with the confidence to come forward to report their own experiences alongside others, as a key barrier to the reporting of sexual victimization is a fear of disbelief (McQueen et al., 2021; Patterson et al., 2009; Sable et al., 2006). By naming suspects of sexual crimes, others with the same experiences at the hands of specific perpetrators may feel more comfortable to come forward due to a ‘safety-in-numbers’ effect. In 2010, the UK government suggested that those accused of rape should be granted anonymity, but as with the earlier Home Affairs Committee recommendation, the plan lacked key empirical data in support of this legal change (Ministry of Justice, 2010). Further, there was no robust comparative data that the stigma suffered by those accused of rape was greater than other types of criminality such as assault, terrorist offenses, or murder, nor how such stigma manifested itself (Rumney & Fenton, 2013). There was also an absence of evidence concerning the impact of not guilty verdicts on public perceptions of those who have been charged. In such an evidential vacuum, policy makers were at risk of introducing a legal reform that was underpinned by faulty reasoning.

### The Current Study

The current study offers a direct test of the assumption that sexual crimes are more heavily stigmatized than other offenses and extends this to consider whether this enhanced stigma effect extends beyond only those with convictions. That is, we seek to explore whether the public will support early notification of an allegation (i.e., pre-trial) to the public. Further, we manipulate the outcome of a trial to test whether the effects of being accused with a sexual offense persists even in the context of a ‘not guilty’ verdict. Our work operationalizes stigma as possessing cognitive and behavioral components, in line with Goffman’s (1963) original conceptualization. As such, we explore attributions of the personalities of people accused of various offenses (a manifestation of the cognitive component of stigma), as well as the degree to which people desire to be socially distanced from them (a manifestation of the behavioral component of stigma). These measures were chosen to be more specific to the target population than other measures would have allowed. For example, we could have adapted stigma scales related to mental illness, or used on the cognitive and behavioral components of measures such as the Attitudes to Sexual Offenders scale (Hogue & Harper, 2019), but these would have lacked the specificity of tailored assessments of specific forms of stigma towards this group. Consistent with the prevailing view that sexual offenses carry a heavy and pervasive level of stigma (see Harper et al., 2017; Willis et al., 2010), we predicted:

Hypothesis 1: Participants will be more supportive of pre-trial public notification of an alleged sex offense, as compared to non-sexual criminal allegations.

Hypothesis 2a: Participants will desire a greater level of social distance from individuals with a history of alleged sexual offending, as compared to those with a history of non-sexual criminal allegations.

Hypothesis 2b: The effect described in Hypothesis 2a will be consistent irrespective of the outcome of a criminal trial.

Hypothesis 3a: Participants will attribute negative personality traits to individuals with a history of alleged sexual offending to a greater degree than they attribute them to those with a history of non-sexual criminal allegations.

Hypothesis 3b: The effect described in Hypothesis 3a will be consistent irrespective of the outcome of a criminal trial.

## Methods

### Design

In this work, we used a fully between-subjects factorial experimental design embedded within an online survey to test our hypotheses. Fundamentally, we have a two-phase design. In the first phase, we manipulate a single factor (labelled ‘criminal allegation’) with four levels: sexual assault against an adult victim, sexual assault against a child victim, physical assault, and theft. At the end of this first phase, we explore the effect of this manipulation on an outcome testing support for civil powers of public notification. In the second phase of the study, we further manipulate a second factor (labeled ‘guilt’) with two levels: guilty and not guilty, equating to the verdict that was reached in response to the alleged offense. We test the effect of this second manipulation on two outcomes related to desires for (1) social distance from the person facing the allegation, and (2) attributions of negative personality traits to them.

### Participants

Individuals were eligible to take part if they were aged over 18 years and declared both residency in the UK and fluency in English. Participants were initially recruited from a range of social networking websites (e.g., local community pages on Facebook and Instagram, and Reddit forums pertaining to UK news and politics). These participants were volunteers and completed for no compensation. We supplemented the sample with students completing our survey in exchange for course credits, and community members enrolled on the Prolific platform. Prolific is a crowdsourcing platform where potential survey participants receive small payments (equivalent to £6 per hour) in exchange for taking part in academic studies. These participants received £0.70 in exchange for complete responses (this equated to around £7 per hour when average completion times were computed). Using such a range of recruitment techniques allowed us to obtain a broader sociodemographic sample than would have been possible if using any one of these methods in isolation. That said, we do not purport to have a sample that is representative of the broader UK population, and caution should still be exercised considering the self-selecting nature of the sample.

An a priori sample calculation using G*Power (v.3.1; Faul et al., 2007) suggested that we would require 231 participants to observe statistical significance for medium-sized effects (*f*^2^ = 0.25, α = .05, power = 90%). In total, 443 participants took part. We removed anybody who declared an age under 18 years (*n* = 1), and only retained participants with complete data on at least one of the outcome measures (see below). This process led to a final sample of 403 participants. This number is above the pre-determined number required, as per our sample calculations. Although we are unable to ascertain exactly how many participants reached the survey via specific social media channels, 114 participants (28.3% of the sample) completed the survey via an institutional research participation platform in exchange for course credits, and 98 participants (24.3% of the sample) took part via Prolific.

The sample was comprised of 213 women and 187 men (three participants did not disclose their sex). The average age was 27.36 years (*SD* = 10.34). The modal qualification level for the sample was an A-level (pre-degree qualification; 39.70%), followed by an undergraduate degree (35.48%). Minorities had a postgraduate degree or doctorate (19.85%), or basic school leaver qualifications or below (4.97%). Politically, 201 participants identified themselves as either ‘left-wing’ or ‘center-left’ (51.01%), 66 participants identified themselves as either ‘right-wing’ or ‘center-right’ (16.67%), and the remaining 128 who answered this question identified themselves as belonging to the political center-ground (32.32%). A total of 281 participants (71.50%) said that they knew somebody who had been the victim of a criminal offense, while only 22 participants (5.56%) said that they personally had a criminal conviction.

### Measures

#### Demographics

We asked participants to provide their age, sex (male vs. female), their highest qualification level, and their political orientation (scored on a 1-5 scale, from ‘left-wing’ to ‘right-wing’). Participants were asked if they knew anybody (including themselves) who had been a victim of crime and, separately, whether the participant themselves had ever been convicted of a criminal offense. Participants also created a unique identification code at this point, which they were asked to remember to allow their data to be removed if requested.

#### Case Vignettes

Four vignettes were developed for the purpose of the current study to examine the role of offense type on levels of stigmatization. Each vignette was approximately 200 words in length and described an alleged offense depicting one of the four target crimes (sexual offense against an adult, sexual offense against a child, physical assault, or theft). In all cases, the perpetrator was male. To reflect typical criminal scenarios, victims were female in the sexual crime vignette, and male in the violent crime vignette. The ‘victim’ in the theft condition was an electronics store. The cases were all described as alleged offenses to permit for the ‘guilt’ variable to be tested successfully. After collecting data in relation to support for pre-trial notification powers (see below), we manipulated the trial outcome by informing participants that the allegation led to either a guilty or not guilty verdict. The full wording of all vignettes is available at https://osf.io/nhvds/?view_only=11501f200bfb40799d086a0c6e467c0a.

#### Support for Police Notification Powers

We asked participants to rate their level of support for police powers to notify various people about the nature of a criminal allegation, specifically in relation to the vignette to which they were assigned. Using a ten-point scale scored from 1 (definitely do not support) to 10 (definitely do support) we asked participants how likely they were to endorse the notification of employers, partners, neighbors, wider family members, and the local media about the alleged offense depicted in their assigned vignette. An average score across the five groups was computed (α = 0.85) with higher scores indicating a greater level of support for pre-trial notification.

#### Desires for Social Distance

We measured participants’ desires for social distance from the individual in their assigned vignette using items from Malinen et al.’s (2014) work on attitudes towards individuals with sexual convictions. Their initial scale used eight items that directly measured desires for social distance (e.g., “How would you like to have [the alleged offender] as your neighbor”). We added three further items to measure anticipatory behavior (e.g., “Would you employ [the alleged offender]?”). Each item (which named the individual in the vignette presented to each participant) was rated on a ten-point scale that was scored from 1 (most definitely not) to 10 (most definitely). We computed an average score across the 11 items as an overall measure of social distance (α = 0.97). Higher scores indicated a greater desire for social distance from the person depicted in the assigned vignette.

#### Personality Attributions

We created a list of 10 adjectives (friendly, nasty, nice, evil, violent, aggressive, kind, horrible, caring, warm) to examine how personality traits were attributed to each of the individuals in our case vignettes. Each adjective was rated using a ten-point scale that was scored from 1 (not at all like this person) to 10 (completely like this person). Items were scored such that high scores equated to higher levels of stigmatization, meaning that all positively-valenced traits were reverse-coded. An average score was computed as an overall measure of stigmatization (α = 0.89).

#### General Punitiveness

We used the General Punitiveness Scale (Maruna & King, 2009) to measure participants’ general levels of punitiveness about criminal justice and sentencing. This is an eight-item measure, with each of these being framed as support for a particular policy or behavior (e.g., “I’d consider volunteering my time or donating money to an organization that supported toughening the sentencing laws in the UK”). Participants were asked to rate each of the items using a six-point scale scored from 1 (strongly disagree) to 6 (strongly agree). An average score was computed (α = 0.77) where higher scores indicated a more punitive stance towards criminal justice.

### Procedure

An advertisement to the survey was posted onto the aforementioned social networking websites and an institutional research participation platform as described previously. Those who were interested in taking part were able to click on the survey link to receive detailed information about the study and the expectations associated with participation. Informed consent was explicitly indicated by clicking a button on this information screen. Although the information page provided details of the study expectation (i.e., that participants would be asked to judge some cases of alleged criminal behavior), the exact hypotheses and experimental conditions were not revealed at this stage.

Participants first provided their demographic information before being randomly assigned to one of four case vignettes. These scenarios were evenly presented across the sample to ensure approximately equal numbers in each condition. Participants then responded to the measure of support for police powers before subsequently being randomized once again to either a ‘guilty’ or ‘not guilty’ verdict condition. Participants then completed the social distance and personality attribution measures in response to their allocated vignette. The general punitiveness scale was then presented at the end of the survey, before participants were fully debriefed. Including this at the end was a deliberate choice owing to the content of the measure. That is, although the statements on this measure refer to broad policy positions that should be unaffected by short crime scenarios, we did not want to prime punitive thinking before the presentation of our experimental vignettes. The debrief information included details about the nature and aims of the study, information on participants’ right to withdraw their data, and contact details for helplines and support websites.

The study was approved by the Nottingham Trent University School of Social Sciences Research Ethics Committee, and followed British Psychological Society ethical codes throughout. An anonymized copy of the survey in .qsf format is available for download at https://osf.io/nhvds/?view_only=11501f200bfb40799d086a0c6e467c0a.

### Data Analysis Plan

We analyzed our data using a series of analyses of variances (ANOVAs). Firstly, we ran a one-way ANOVA examining the effect of offense allegation on support for the police sharing this information with those who may have an interest in hearing about it (e.g., family and employers). Following this, we ran a 4 (‘criminal allegation’: adult-directed sexual offense, child-directed sexual offense, violent offense, acquisitive offense) × 2 (‘guilt’: guilty, not guilty) ANOVA on participants’ levels of stigmatization of the individual about whom they read. Separate analyses were run for stigmatization in relation to desires for social distance and negative personality perceptions. All data and code are available for download at https://osf.io/nhvds/?view_only=11501f200bfb40799d086a0c6e467c0a.

All analyses were ran with and without general punitiveness as a covariate. Controlling for punitiveness did not affect the overall pattern of results, and we report only the controlled analyses here. All between-groups contrasts within our ANOVA reporting are Tukey-corrected *p*-values. Uncontrolled models can be verified using the datafile and code available at https://osf.io/nhvds/?view_only=11501f200bfb40799d086a0c6e467c0a.

## Results

### Randomization Check

Before running any analyses, we checked to ensure that our randomization procedures produced conditions that contained participants with equal levels of general punitiveness by running the 4 (criminal allegation) × 2 (guilt) ANOVA model on scores collected from the General Punitiveness Scale. We found no effects of either criminal allegation (*F*(3, 379) = 1.87, *p* = .135 η^2^_g_ = 0.01) or guilt (*F*(1, 379) = 0.03, *p* = .858, η^2^_g_ < 0.01), nor an interaction between these variables (*F*(3, 379) = 0.69, *p* = .561, η^2^_g_ < 0.01). As such, we concluded that randomization was successful as none of the individual experimental conditions contained participants who were excessively punitive on a general level.

Scale coding was carried out using SPSS, while data analysis and visualization was conducted using R (R Core Team, 2021). For clarity, we present estimated marginal means for all conditions in each analysis in Table 1.

**Table 1.** Estimated marginal means, by outcome and condition.

Criminal Allegation	Support for Police Notification Powers	Desired Social Distance		Stigmatizing Personality Attributions	
		Not Guilty	Guilty	Not Guilty	Guilty
Adult sex offense	4.10 (0.21)	6.19 (0.17)	7.64 (0.17)	6.19 (0.17)	7.64 (0.17)
Child sex offense	5.45 (0.22)	5.60 (0.17)	7.99 (0.17)	5.85 (0.17)	6.62 (0.17)
Assault	3.96 (0.21)	5.85 (0.17)	6.62 (0.17)	5.60 (0.17)	7.99 (0.17)
Theft	3.81 (0.22)	4.88 (0.17)	5.98 (0.18)	4.88 (0.17)	5.98 (0.18)

*Note.* Data represent estimated marginal means (controlling for general punitiveness) as computed using the *emmeans* package (Lenth et al., 2021), with ±1 *SE* in parentheses. All outcomes range from 1–10, with higher scores indicating greater support for community notification, desired social distance, or stigmatization.

### Support for Police Notification Powers

We found a significant main effect of ‘criminal allegation’ on support for police notification powers, *F*(3, 381) = 12.31, *p* < .001, *η*^2^_g_ = 0.09. General levels of punitiveness were also associated with greater levels of support for police notification, *F*(1, 381) = 15.65, *p* < .001, *η*^2^_g_ = 0.04. Exploring the estimated marginal mean differences between the conditions, we found a significant difference in notification support in relation to the individual accused of a child sex offense and all other conditions:

• Adult sex offense: *M*_diff_ = 1.35, 95% CI [0.57, 2.21], *p* < .001
• Assault: *M*_diff_ = 1.50, 95% CI [0.72, 2.28], *p* < .001
• Theft: *M*_diff_ = 1.64, 95% CI [0.84, 2.43], *p* < .001

**Figure 1.**
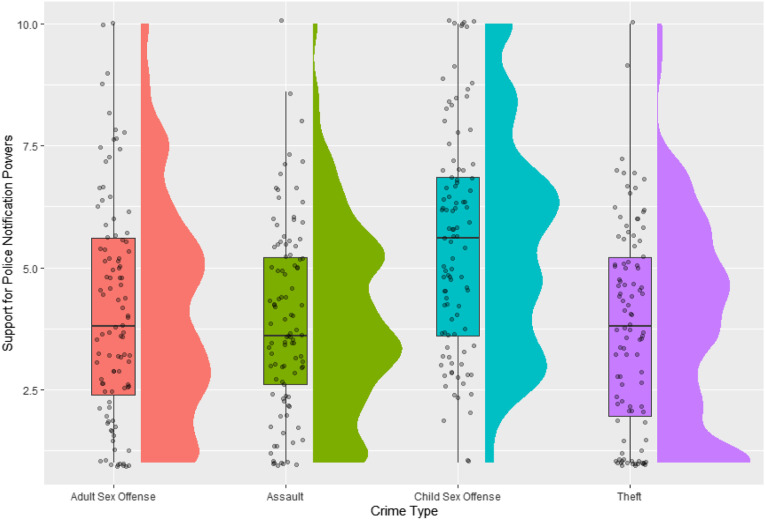
Raincloud plot of the data for support for police notification powers, by crime type. Distributions plot the overall patterns of the data for each allegation type, while the boxplots represent the mean and interquartile range of scores. Dots represent individual datapoints within the sample.

No other conditions significantly differed from each other (*p*s ≥ .776). A graphical presentation of the data is presented in Figure 1.

### Conviction Status and Stigmatization

#### Desires for Social Distance

**Figure 2.**
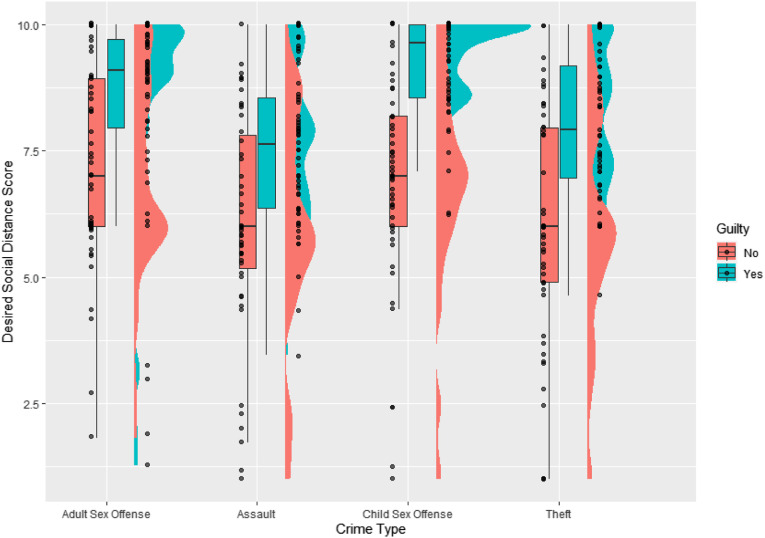
Raincloud plot of the data for desired social distance, by crime type and verdict. Distributions plot the overall patterns of the data for each allegation type, while the boxplots represent the mean and interquartile range of scores. Dots represent individual datapoints within the sample.

We found a significant main effect of ‘criminal allegation’ on desired social distance, *F*(3, 378) = 5.21, *p* = .002, *η*^2^_g_ = 0.07. There was also a significant main effect of ‘guilt’, *F*(1, 378) = 8.09, *p* = .005, *η*^2^_g_ = 0.19. However, there was no interaction between these variables, *F*(3, 378) = 2.58, *p* = .053, *η*^2^_g_ = 0.02. General levels of punitiveness were significantly associated with greater levels of desired social distance, *F*(1, 378) = 17.55, *p* < .001, *η*^2^_g_ = 0.04. These data are plotted in Figure 2.

Examining the significant main effect for criminal allegation, we found that those accused of sexual offenses were more subject to more desired social distance than those who had faced allegations of either assault (adult sexual offense: *M*_diff_ = 0.97, 95% CI [0.30, 1.64], *p* = .001; child sex offense: *M*_diff_ = 1.15, 95% CI [0.47, 1.83], *p* < .001) or theft (adult sexual offense: *M*_diff_ = 0.83, 95% CI [0.15, 1.51], *p* = .010; child sex offense: *M*_diff_ = 1.02, 95% CI [0.33, 1.71], *p* = .001). Desired social distance from those who had faced allegations of adult sex offenses did not significantly differ to desires to be distant from those accused of child sexual offenses, *M*_diff_ = −0.18, 95% CI [-0.86, 0.50], *p* = .899. Participants were also equally likely to express a desire to be distant from those accused of theft compared to assault, *M*_diff_ = 0.13, 95% CI [-0.55, 0.82], *p* = .958.

In relation to the main effect of guilt, participants expressed more desire to be socially distant from those found guilty than those found not guilty, *M*_diff_ = 1.75, 95% CI [1.37, 2.11], *p* < .001.

#### Attributions of Malevolent Personality Traits

**Figure 3.**
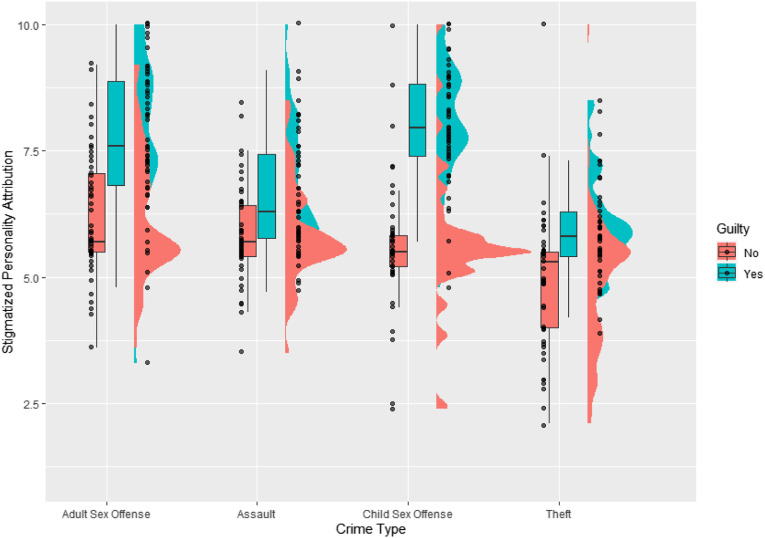
Raincloud plot of the data for stigmatizing personality attributions, by crime type and verdict. Distributions plot the overall patterns of the data for each allegation type, while the boxplots represent the mean and interquartile range of scores. Dots represent individual datapoints within the sample.

We found a significant main effect of ‘criminal allegation’ on stigmatizing personality attributions, *F*(3, 376) = 10.63, *p* < .001, η^2^_g_ = 0.22. There was also a significant main effect of ‘guilt’, *F*(1, 376) = 37.88, *p* < .001, η^2^_g_ = 0.27. A significant interaction was also present between these variables, *F*(3, 376) = 8.03, *p* < .001, η^2^_g_ = 0.06. General levels of punitiveness were significantly associated with greater levels of stigmatization, *F*(1, 376) = 14.05, *p* < .001, η^2^_g_ = 0.03. Figure 3 presents these data.

Examining the main effect of criminal allegation, all between-condition comparisons were statistically significant, save for the comparison between the two sexual offense categories. In practical terms, those facing sexual offense allegations were more stigmatized than those facing either assault or theft allegation, and those facing assault allegation were more stigmatized than those accused of theft:

• Adult vs. child sexual offense: *M*_diff_ = −0.16, 95% CI [-0.28, 0.59], *p* = .791
• Adult sexual offense vs assault: *M*_diff_ = 0.69, 95% CI [0.26, 1.12], *p* < .001
• Adult sexual offense vs theft: *M*_diff_ = 1.59, 95% CI [1.14, 2.03], *p* < .001
• Child sexual offense vs assault: *M*_diff_ = 0.53, 95% CI [0.09, 0.97], *p* = .010
• Child sexual offense vs theft: *M*_diff_ = 1.43, 95% CI [0.99, 1.88], *p* < .001
• Assault vs theft: *M*_diff_ = 0.90, 95% CI [0.46, 1.34], *p* < .001

Examining the main effect of guilt, those who were found guilty were more heavily stigmatized than those found not guilty, *M*_diff_ = 1.42, 95% CI [1.18, 1.65], *p* < .001.

Unpacking the significant interaction, we found different trends of stigmatizing attitude attributions as a function of criminal allegation among the ‘not guilty’ and ‘guilty’ conditions. When a not guilty verdict was reached, lower stigmatizing attributions were made about the individual accused of theft than in comparison to all other allegation conditions:

• Adult sex offense: *M*_diff_ = −1.66, 95% CI [-2.30–1.03], *p* < .001
• Child sex offense: *M*_diff_ = −0.72, 95% CI [-1.34, −0.09], *p* = .017
• Assault: *M*_diff_ = −0.96, 95% CI [-1.59, −0.34], *p* = .001

All other between-condition comparisons were not statistically significant (*p*s ≥ .068), indicating that crime type did not influence the level of stigmatizing personality attributions following a not guilty verdict. In comparison, all between-condition comparisons were statistically significant if the trial outcome was a guilty verdict, save for the comparison between the two sexual offense categories. Those facing sexual offense allegations were subject to more stigmatized personality attributions than those facing either assault or theft allegations, and those facing assault allegations were more stigmatized than those accused of theft:

• Adult vs. child sexual offense: *M*_diff_ = −0.34, 95% CI [-0.96, 0.28], p = .485
• Adult sexual offense vs assault: *M*_diff_ = 1.02, 95% CI [0.42, 1.63], *p* < .001
• Adult sexual offense vs theft: *M*_diff_ = 1.66, 95% CI [1.03, 2.30], *p* < .001
• Child sexual offense vs assault: *M*_diff_ = 1.37, 95% CI [0.75, 1.98], *p* < .001
• Child sexual offense vs theft: *M*_diff_ = 2.01, 95% CI [1.37, 2.65], *p* < .001
• Assault vs theft: *M*_diff_ = 0.64, 95% CI [0.01, 1.27], *p* = .045

## Discussion

In this work, we sought to examine the roles of criminal allegation and verdict on support for pre-trial public notifications, and on levels of social stigma. We found partial support for Hypothesis 1, in that participants expressed a greater degree of support for the pre-trial disclosure by police of accusations about child sex offenses. This means that participants were more supportive of family, employers, and the local media being told about this accusation, before a trial had taken place, than when the accusation was either a sex offense committed against an adult, a physical assault, or theft. However, we found no evidence of increased levels of support for police-initiated public notifications about allegations of sex offenses committed against adult victims. This is particularly interesting owing to the burgeoning literature on ‘attitudes towards sexual crime’ which, in taking such a broad approach, may be missing a significant degree of nuance. That is, punitive views about sexual crime might be triggered by intuitive availability- or representativeness-related heuristics that bring to mind child-directed crimes (see Harper & Hogue, 2014, 2017). This was also highlighted by Harris and Socia (2016) who argued that:

Prompts such as “What percentage of sex offenders do you think commit new sexual crimes after their release from prison?” or “Do you think that the names and addresses of convicted sex offenders should be made available to the public?” implicitly force respondents to make general inferences and statements about a knowingly diverse population. Ultimately, it may be that the resulting research tells us more about respondents’ visceral reactions to the “sex offender” and “JSO” [“juvenile sex offender”] labels than it does about rational assessments regarding adults or youth who have perpetrated sexual offenses (p. 661).

Although we did not test support for formal community notification procedures that are tied to registration policies (see Connor & Tewksbury, 2017; Kernsmith et al., 2009; Levenson et al., 2007) public attitudes are important drivers of social policy related to sexual crime (Harper & Hogue, 2014; Willis et al., 2010). The results of this study suggest a level of support for (or a desire for) notifications to be made by law enforcement officers about sexual crime allegations involving child victims even before a conviction has been achieved. There are several potential reasons for this. First, it might be that there is simply a voyeuristic aspect to simply wanting to know about sexual allegations that is not present when there are allegations associated with other criminal categories. This is consistent with a view that suggests how criminal justice news often becomes tied up with entertainment and the rise of the *celebrity sex offender* (Harper & Hogue, 2014; Madoc-Jones et al., 2014). On the contrary, it could be that notifications about child sexual abuse allegations provide community members with a sense of safety, in that they feel more empowered to make arrangements to protect children (and those of friends and family) from a perceived threat (Bandy, 2011). This has been reported as a key driver of public support from notification procedures regarding individuals who are registered as committing sexual offenses (Beck & Travis, 2004; Caputo & Brodsky, 2004; Connor & Tewksbury, 2017), but to our knowledge this is the first demonstration of this potential trend at the pre-conviction and pre-registration stage of criminal proceedings.

There was support for both Hypothesis 2a and 2b. That is, those accused of sexual offenses (both in relation to adult and child victims) were subject to greater levels of behavioral stigmatization in the form of participants’ desire to socially distance from them, as compared to those accused of the non-sexual crimes of physical assault and theft (Hypothesis 2a). Further, there was no statistically significant interaction between the criminal allegation and the trial verdict in relation to desired social distance, meaning that this effect of allegation was consistent irrespective of whether the individuals were found guilty (Hypothesis 2b). Although it should be highlighted that the significant main effect of ‘guilt’ does suggest that a not guilty verdict attenuated the negative effects of a sexual offense allegation on desired social distance, participants did still discriminate between those who faced these allegations and those who were accused of non-sexual offenses. There are links here to the anonymity debate, wherein it has been suggested that sexual crime allegations carry a unique level of (increased) stigma in comparison to the other criminal offences featured in this study. Given the links between negative social attitudes about those with sexual convictions and long-term outcomes for those who are subject to them, including informal restrictions on residency, poorer quality personal relationships, and difficulties with employment (Göbbels et al., 2012; Harper et al., 2017; Willis et al., 2010), it is plausible to argue that stigma could inhibit positive outcomes for both those acquitted of sexual offense allegations (in relation to social relationships) and those who are criminally convicted (in terms of reintegration and desistance). Positive psychological frameworks such as the Good Lives Model (Ward et al., 2007) or Circles of Support and Accountability (Dwerryhouse et al., 2020) have been applied to the rehabilitation of people convicted of sexual offences in relation to assisting with long-term desistance from crime (see Göbbels et al., 2012). In this framework, affording such groups the opportunity to rebuild their lives and their identities through work, education, and forming authentic interpersonal relationships is the key to both emotional wellbeing and preventing future re-offending. However, there is an acknowledgement that enabling the pursuit of such goals using a positive psychological approach is also a constructive route to mental wellness universally (Maddux, 2008; Ryff & Keyes, 1995; Seligman, 2019). Such pursuits are made more difficult when confronted by stigma (Göbbels et al., 2012), which may partly explain the high rates of suicide among those accused and convicted of sexual offenses in comparison to other criminal allegations (Key et al., 2021). The criminal allegation data discussed above provide support for the current police practice of not naming criminal suspects prior to charge (College of Policing, 2021). The findings related to conviction status also lend some weight to the argument that sex offense defendants might have their identity legally protected up to the point of conviction. This specific proposal, however, has to be weighed against other policy arguments. For example, this practice would treat sexual offense defendants differently from other defendants accused of crimes such as murder and, in some instances, prevent new complainants from reporting to the police due to the absence of pre-trial publicity (McGlynn, 2011; Rumney & Fenton, 2013).

We found mixed support for Hypotheses 3a and 3b. Stigmatizing personality attributions (operationalized as attributing negative traits to the individual within a given participant’s assigned vignette) were higher for those depicted in vignettes where the criminal allegation was sexual in nature (whether this involved an adult or a child victim) compared to either of the non-sexual allegations (Hypothesis 3a). However, this effect was not consistent across both levels of guilt. That is, only the theft condition led to significantly reduced levels of stigmatization when the trial verdict was given as not guilty, but the main effect pattern (higher levels of stigmatization being directed to those with sexual offense allegations) held when the defendant was found guilty (Hypothesis 3b). These data are positive in some sense, as they highlight that lay members of the public do not judge a person by the allegations made against them independent of the conviction decision. They also support an expected trend wherein people do not judge people according to a ‘criminal schema’ but do make judgments in line with the perceived severity of the crimes for which they are convicted.

### Limitations and Future Directions

The study is limited by the artificial nature of the stimuli used. That is, we constructed the vignettes for the purposes of this study, and they were not reflective of any specific case that has occurred. There were also some important differences between our vignettes, which we felt were necessary to preserve the ecological validity of the scenarios but may nonetheless have affected the data. For example, our violence vignette involved a male perpetrator assaulting a male victim, while both sexual vignettes involved a male perpetrator and female victim. Replications might use a broader range of scenarios to control for the gendered nature of the offences used. They might also explore the relationship between such factors with outcomes such as believability or plausibility, which we did not measure for our vignettes.

In addition, participants may have inferred the artificiality of the scenarios due to our presentation of the vignettes (i.e., plain text within the experimental survey), and may have responded differently to these cases than they would to ongoing offense allegations. Future work might address this limitation in multiple ways. The most obvious of these would be to use real cases as stimuli. This can be achieved by using experience sampling techniques to monitor social responses to individual cases as they develop. That is, individuals might be surveyed within days of a story emerging about a given case, and then slightly before the trial, and then again once a verdict has been reached. Although this method may be ideal for addressing our question, it poses some important logistical challenges, not least being ready to collect data (i.e., with ethical approvals granted and materials ready) in a way that is reactive to the 24-hour news cycle, and subsequently recruiting a sample for multiple waves of data collection. An alternative method might be to present the stimuli as news stories using mock newspaper articles or online news pages. This may help to overcome the possibility of participants inferring the artificiality of the stimuli without needing to collect data about ongoing cases in real time.

From a sampling perspective, it is worth noting that we recruited participants from a range of sources to improve the potential for diversity in our sample. That is, we wanted to avoid a situation whereby we only had paid participants from crowdsourced platforms, university participation schemes for students, or volunteers on social media. In doing so, however, we are left unclear about whether participants engaging with the survey through these different channels were qualitatively different, and whether there may be cohort effects within the data. We did not have sufficient statistical power to run separate analyses on subgroups of participants, but future work might look into this possibility. If it is found that results are consistent across participants recruited through different services and platforms, this opens up the possibility to maximize sample sizes at a lower cost to researchers, while maintaining data quality.

We are also aware that many people in our sample (72%) knew of somebody who had been the victim of a crime. We do not know the proportions of these that are attributable to different crime types, and so it is plausible that the type of crime victim to which one is acquainted could affect our results. In American surveys, slightly less than 50% reported knowing somebody who had been victims of sexual crimes (Rydberg et al., 2018; Socia et al., 2019). There have been some studies wherein knowing a victim of sexual offending, or having been the victim directly, has been associated with more punitive attitudes towards people with sexual convictions (e.g., Socia et al., 2019), while others have reported more positive attitudes (e.g., Spoo et al., 2018). A review of the literature suggested that this variable is generally unrelated to attitudes, as, for many, perpetrators are known to them, meaning that intuitive responses related to social stereotypes tied to the ‘sex offender’ label are disrupted (see Harper et al., 2017). Nonetheless, it is possible that this higher rate of knowing a victim of sexual crime might raise punitive responses to this crime type (in a manner that is consistent across conditions, owing to the randomization of participants to different scenarios). Although we are confident in the data, owing to the review evidence about the effect of this variable on attitudes, readers might reflect on this issue when interpreting our findings. This may be particularly important in our sample where people know victims whose perpetrators have not been brought to justice. Exploring these specific issues in large nationally-representative samples would be an interesting avenue for future work.

There are widely acknowledged issues with between-groups analyses, such as a loss of statistical power and issues with randomization leading to psychologically unequal groups. We attempted to mitigate some of these limitations by conducting an a priori power analysis to achieve the required statistical power, recruiting a sample above this number, and conducting a randomization check to ensure that no individual condition was comprised of excessively punitive participants. Nonetheless, it is not possible for us to say that participants would have responded in the same manner as our broad pattern suggests if they had been exposed to all stimuli in a within-groups design.

In only using four vignettes (one per criminal allegation) we may have unintentionally embedded bias within the scenarios presented to participants. Future work might look to develop a suite of criminal allegation vignettes that have been comprehensively pre-tested on such indices to ensure that researchers can make informed decisions about their selection of experimental stimuli in subsequent studies (see Chester & Lasko, 2021). These should include a wider range of offense categories than was feasible to include within the current work, and explore response to terror offenses, hate crimes, and more serious violent offending accusations. One way to overcome the limitations of single-study designs and to accelerate the pace of such tool development would be to engage in consortia-led research, whereby many labs and research teams work collaboratively to collect data at the individual lab level before pooling this into a large, and preferably international, dataset. From this approach it is possible to gather large quantities of comparative data in a relatively time- and resource-efficient manner.

## Conclusions

This work has sought to address a gap in the literature in relation to the comparative levels of stigma directed towards those accused of sexual and non-sexual offenses, and whether such stigmatization is dependent upon whether a conviction is handed down. We found support for the conclusion that sexual offense allegations (and convictions) are more heavily stigmatized than allegations related to other crime types, and that this was particularly the case when allegations involved child victims. Receiving a ‘not guilty’ verdict did attenuate the level of stigma directed towards all crime types, though this effect was largest in relation to sexual offense allegations. In practical terms, those found ‘not guilty’ of a sexual offense were no more or less stigmatized, from a statistical perspective, than those found cleared of non-sexual allegations. However, across the board we found evidence of stigmatization (operationalized as average stigma levels around the mid-point of the scale) irrespective of a non-guilty verdict. Based on these data, there is an argument to be made that those accused of crimes might be well-served by a right to anonymity prior to conviction, with identities being released upon a guilty verdict. However, more work is required to consider the issue of the anonymity, including the utility of the police revealing a suspect’s identity when investigating a sexual crime. This is not an easy debate to balance, with the pursuit of justice such cases (for victims who may come forward in response to named reports of criminal charges before prosecution) needing to be considered alongside the legal rights of suspects to be assumed innocent until proven guilty. Nonetheless, this first look at the comparative stigmatization of those with and without convictions after facing a sexual crime allegation provides a strong basis for future research to build on.
